# Differential predictors of smoking quit attempts between single- and poly-tobacco-using adolescents: a Korean national survey analysis

**DOI:** 10.3389/fpubh.2026.1727685

**Published:** 2026-04-07

**Authors:** Eunkyo Kang, Hyewon Lee, Juyoung Choi, Hyorim Ju

**Affiliations:** 1 National Cancer Control Institute, National Cancer Center, Goyang, Republic of Korea; 2 National Cancer Center Graduate School of Cancer Science and Policy, National Cancer Center, Goyang, Republic of Korea; 3 Department of Family Medicine, National Cancer Center, Goyang, Republic of Korea; 4 Department of Family Medicine, Dankook University Hospital, Cheonan, Chungcheongnam-do, Republic of Korea; 5 Department of Family Medicine, Seoul National University College of Medicine, Seoul, Republic of Korea

**Keywords:** adolescents, poly-tobacco use, predictors, quit attempts, risk factors, single-tobacco use

## Abstract

**Background:**

Despite the rapid rise in adolescent poly-tobacco use, evidence on what drives quit attempts in this high-risk group remains limited compared to single-product users. This study identifies these differential predictors using a socio-ecological framework to inform tailored tobacco control strategies.

**Methods:**

This study performed a cross-sectional analysis using complex sample logistic regression on pooled data from the 2023–2024 Korea Youth Risk Behavior Survey (KYRBS), a nationwide survey conducted in South Korea. Participants included a total of 5,127 current adolescent smokers, of whom 40.8% were single-product users and 59.2% were poly-tobacco users. The primary outcome was self-reported quit attempts within the past 12 months. Predictors included user type and individual, interpersonal, organizational, and community/policy factors based on the socio-ecological model.

**Results:**

Poly-tobacco users exhibited a more vulnerable profile but reported significantly more quit attempts than single-product users. While school-based education and peer smoking were shared facilitators, the positive impact of education was significantly more pronounced in poly-tobacco users (*p* < 0.001). Notably, anti-smoking media campaigns were associated with quit attempts exclusively among poly-tobacco users (aOR = 1.29, 95% CI [1.09–1.52]), showing no significant effect on single-product users.

**Conclusion:**

Predictors of adolescent quit attempts differ significantly by tobacco use complexity. Although a higher-risk group, poly-tobacco users showed a stronger association between quit attempts and both school-based education and media campaigns. This highlights the need for tailored tobacco control strategies that account for varying levels of receptivity among different user groups.

## Introduction

Tobacco use among adolescents remains a critical public health challenge, serving as a primary gateway to long-term morbidity and mortality (1). The challenge has changed significantly due to the rise of novel tobacco products, including e-cigarettes (ECs) and heated tobacco products (HTPs) (2). Consequently, poly-tobacco use, or the concurrent use of multiple tobacco products, has emerged as one of the patterns among youth smokers (2–4). This shift presents a formidable public health problem, as poly-tobacco users typically exhibit higher levels of nicotine dependence, more substance use comorbidities, and lower intentions to quit compared to their single-product-using peers (5, 6). Understanding the dynamics of cessation in this new era is crucial for effective tobacco control.

An adolescent’s decision to quit smoking involves more than just willpower; it is influenced by various interconnected factors (7, 8). The socio-ecological model provides a robust theoretical framework for understanding this complexity, positing that health behaviors are determined by the dynamic interaction of factors at the individual, interpersonal, organizational, and community/policy levels (9). Evidence demonstrates the importance of factors across all ecological levels. Individual-level factors, such as the degree of nicotine dependence and mental health status (e.g., stress, depression), are powerful predictors of cessation outcomes (10, 11). At the interpersonal level, exposure to smoking within family and peer networks significantly undermines cessation efforts, as supported by previous studies (10, 12). Concurrently, organizational and policy-level factors, such as school-based prevention programs, national anti-smoking media campaigns, pictorial warning labels, and various interventions, including telephone counseling and digital tools, can effectively facilitate quit attempts (13–17).

Despite a substantial body of research, a critical knowledge gap persists (18). Previous studies have focused on adolescents who smoke conventional cigarettes (CCs), and it remains uncertain whether these findings are generalizable to the growing population of poly-tobacco users (19). Interventions designed for single-product smokers may not resonate with poly-tobacco users, who have different motivations, risk perceptions, and patterns of dependency (20, 21). For instance, a traditional school-based message that “all tobacco is harmful” could induce cognitive dissonance in an adolescent who initiated e-cigarette use as a perceived harm-reduction strategy, potentially diminishing the intervention’s effectiveness (22, 23). While some research has identified factors that differentiate single- from multiple-product users, these studies have primarily focused on use escalation rather than cessation dynamics (3, 24).

This gap in the literature highlights the urgent need for research on the differential predictors of quit attempts between single- and poly-tobacco users (2, 25). The distinct psychological profiles and social contexts of these groups may lead to different factors influencing their motivation to quit. Interpersonal influences, like peer networks, are universally important. However, policy-level interventions, such as media campaigns, may have a greater impact on poly-tobacco users, who engage more with diverse product marketing and social norms (26). Understanding these differences is crucial for advancing beyond a uniform approach to adolescent tobacco control (27).

Consequently, the current absence of evidence-based insights regarding cessation predictors for poly-tobacco users represents a critical public health problem, as it prevents the design of effective interventions for this high-risk group. To address this issue, we utilized large-scale, nationally representative data from the 2023 and 2024 Korea Youth Risk Behavior Survey (KYRBS). Guided by the socio-ecological model, this study aimed: (1) to identify and compare multi-level predictors of smoking quit attempts among Korean adolescent smokers, and (2) to determine the extent to which these influences vary between single- and poly-tobacco users. Based on the existing literature, we tested the following hypotheses: (H1) Socio-ecological factors across all levels will be associated with quit attempts in both groups; and (H2) the positive associations between quit attempts and institutional/community factors (school education and media campaigns) will be significantly more pronounced for poly-tobacco users than for single-product users. By elucidating these differential effects, this study seeks to provide actionable, evidence-based insights for developing tailored cessation strategies that can effectively reach all segments of the adolescent smoking population.

## Method

### Data source and study design

The primary objective of this study was to identify and compare the multi-level predictors of smoking quit attempts among Korean adolescent smokers, with a specific focus on differentiating between single- and poly-tobacco users. This study was a secondary analysis of cross-sectional data, utilizing pooled raw data from the 19th (2023) and 20th (2024) Korea Youth Risk Behavior Survey (KYRBS), a nationally representative survey conducted by the Korea Disease Control and Prevention Agency (KDCA) (28). The KYRBS employs a stratified multi-stage cluster sampling design to ensure a representative sample of the adolescent population in the Republic of Korea. The target population comprises all middle and high school students, surveyed annually through an anonymous, self-administered online questionnaire to maximize response validity. Informed consent was obtained from all student participants. To ensure data reliability, the KDCA implements a rigorous quality assurance protocol, including real-time logic checks to prevent inconsistent responses and subsequent data cleaning guided by an expert committee to verify the internal consistency and biological plausibility of the data. The KDCA approved the original survey protocol, and this study’s publicly available dataset includes de-identified data. The combined dataset from 2023 and 2024 included 107,533 participants from 800 sampled schools each year. From this population, our final analytical sample was restricted to respondents defined as “current smokers”—those who reported using at least one of three tobacco products (CCs, ECs, or HTPs) on one or more days within the past 30 days. The study obtained approval for the use of raw data from the KDCA and was reviewed and approved by the Institutional Review Board of National Cancer Center (IRB No. NCC2023-0167). We followed the STROBE reporting guidelines for observational studies.

### Variable measurement and operational definition

Variables for this study were structured according to the multi-level framework of the socio-ecological model, encompassing individual, interpersonal, organizational, and community/policy domains. The primary dependent variable, “smoking quit attempt,” was operationalized as a dichotomous variable based on self-reported attempts to quit smoking within the past 12 months. To ensure comparability with international surveillance standards, “current use” for each tobacco product was defined as using the product on one or more days within the past 30 days. Accordingly, the key independent and moderator variable, “tobacco user type,” was categorized by combining these frequency-based responses regarding the use of CCs, ECs, and HTPs into two groups: “single-product users” (defined as meeting the current use threshold for only one product type) and “poly-tobacco users” (defined as simultaneously meeting the threshold for two or more product types).

Predictor variables were defined as follows: Individual-level factors included gender, school grade, and academic performance (measured by the question “How has your academic performance been over the past 12 months?” using a 5-point Likert scale: 1 = high, 2 = above average, 3 = average, 4 = below average, and 5 = low). Perceived household economic status was also measured on a similar 5-point Likert scale. Perceived stress was assessed with the question “How much stress do you usually feel in your daily life?” with responses on a 5-point scale ranging from “very much” to “not at all”; for analysis, these were dichotomized into “high” (combining “very much” and “a lot”) and “low” (combining “a little,” “not much,” and “not at all”) categories. Experience of depressive mood (in the past 12 months) was treated as a dichotomous variable.

Interpersonal-level factors comprised the presence of smoking family members, the smoking status of close friends, and the frequency of exposure to secondhand smoke within the home in the past week. The organizational-level factor was defined by experience with school-based smoking prevention education in the past 12 months. Finally, community/policy-level factors included exposure to anti-smoking media campaigns within the past 30 days, perceived ease of access to tobacco products (measured on a 5-point scale), and exposure to tobacco advertisements.

### Statistical analyses

To generate nationally representative estimates and adjust for the multi-stage cluster sampling design, all statistical analyses were performed using a complex sample design. This approach incorporates sampling weights, stratification variables, and primary sampling units (PSUs) to account for the design effect and the intraclass correlation inherent in data sampled at the school level, thereby providing more accurate standard errors and point estimates. They were conducted in four sequential stages. First, to describe the sample and compare baseline characteristics between single- and poly-tobacco user groups, Rao–Scott chi-square tests for categorical variables and independent t-tests for continuous variables were conducted. Second, to identify the predictors of smoking quit attempts among the total sample of adolescent smokers, a hierarchical multivariable logistic regression was performed. Predictor variables were entered in blocks corresponding to the levels of the socio-ecological model (Model 1: Individual → Model 2: +Interpersonal → Model 3: +Organizational & Community/Policy) to assess the incremental explanatory power of each level, with model fit evaluated using the Nagelkerke R^2^ statistic. Third, to formally test the moderating effect of tobacco user type, interaction terms (e.g., user type × school-based education; user type × perceived stress) were introduced into the final regression model. The results of these stratified models were visualized using a forest plot to intuitively compare the adjusted odds ratios (aORs) of key predictors between the two groups.

To supplement the primary analyses, several supplementary visualizations and exploratory analyses were also conducted. As part of the initial descriptive phase, bar graphs were generated to visualize quit attempt rates across more granular user typologies (e.g., single conventional, dual user, triple user) and to illustrate the distribution of product combinations within the poly-tobacco user group. Following the interaction analysis, a significant moderation effect was graphically represented using an interaction plot to visualize the differential effect of predictors, such as school-based education, on the predicted probability of a quit attempt for each user group. Statistical significance was set at a two-tailed *p*-value of <0.05, and all analyses were conducted using Python (Version 3.9) with the “statsmodels” and “pandas” libraries.

## Results

### Baseline characteristics of participants by tobacco use pattern

The final analytical sample comprised 5,127 current adolescent smokers, categorized into single-product (40.8%) and poly-tobacco users (59.2%). Bivariate analyses showed that these two groups differed significantly across nearly all psychological, social, and environmental characteristics, with the exception of gender distribution (Table 1).

**Table 1:** Baseline characteristics of participants by tobacco use pattern.

Variable		Overall, *N* (%)	Single users, *N* (%)	Multi users, *N* (%)	*p*-value
Sex	Male	3,311 (64.6%)	1,352 (64.6%)	1959 (64.6%)	N/S
	Female	1816 (35.4%)	742 (35.4%)	1,074 (35.4%)	
Academic record	High	412 (8.0%)	178 (8.5%)	234 (7.7%)	0.008
	Above average	832 (16.2%)	351 (16.8%)	481 (15.9%)	
	Average	1,277 (24.9%)	535 (25.5%)	742 (24.5%)	
	Below average	1,434 (28.0%)	606 (28.9%)	828 (27.3%)	
	Low	1,171 (22.8%)	424 (20.2%)	747 (24.6%)	
Perceived stress	Very frequently	930 (18.1%)	319 (15.2%)	611 (20.1%)	< 0.001
	Frequently	1,579 (30.8%)	642 (30.7%)	937 (30.9%)	
	Sometimes	1840 (35.9%)	812 (38.8%)	1,028 (33.9%)	
	Rarely	590 (11.5%)	249 (11.9%)	341 (11.2%)	
	Never	188 (3.7%)	72 (3.4%)	116 (3.8%)	
Depressive feelings	No	2,707 (52.8%)	1,172 (56.0%)	1,535 (50.6%)	< 0.001
	Yes	2,420 (47.2%)	922 (44.0%)	1,498 (49.4%)	
Quit attempt (past year)	No	1819 (35.5%)	805 (38.4%)	1,014 (33.4%)	< 0.001
	Yes	3,308 (64.5%)	1,289 (61.6%)	2019 (66.6%)	
Family smoker present	No	3,607 (70.4%)	1,507 (72.0%)	2,100 (69.2%)	0.038
	Yes	1,520 (29.6%)	587 (28.0%)	933 (30.8%)	
Friends who smoke (Any)	No	2,918 (56.9%)	1,272 (60.7%)	1,646 (54.3%)	< 0.001
	Yes	2,209 (43.1%)	822 (39.3%)	1,387 (45.7%)	
Secondhand smoke at home (Any)	No	3,389 (66.1%)	1,471 (70.2%)	1918 (63.2%)	< 0.001
	Yes	1738 (33.9%)	623 (29.8%)	1,115 (36.8%)	
School-based smoking education	No	3,416 (66.6%)	1,446 (69.1%)	1970 (65.0%)	0.002
	Yes	1711 (33.4%)	648 (30.9%)	1,063 (35.0%)	
Anti-tobacco media exposure	No	2,708 (52.8%)	1,143 (54.6%)	1,565 (51.6%)	0.038
	Yes	2,419 (47.2%)	951 (45.4%)	1,468 (48.4%)	
Easy access to buy tobacco	No	3,084 (60.2%)	1,516 (72.4%)	1,568 (51.7%)	< 0.001
	Yes	2043 (39.8%)	578 (27.6%)	1,465 (48.3%)	

NS, not significant. % indicates weighted percentages.

Poly-tobacco users demonstrated a profile of greater vulnerability, reporting significantly lower academic performance (*p* = 0.008), higher levels of perceived stress (*p* < 0.001), and more frequent depressive feelings (*p* < 0.001). Their environment was also more conducive to smoking; they were more likely to have family (*p* = 0.038) and friends (*p* < 0.001) who smoke, experience secondhand smoke at home (*p* < 0.001), and reported substantially easier access to purchasing tobacco (*p* < 0.001). Interestingly, despite this higher-risk profile, poly-tobacco users also reported a significantly higher rate of quit attempts in the past year (*p* < 0.001), suggesting a complex interplay between risk behaviors and a desire to quit.

**Figure 1:**
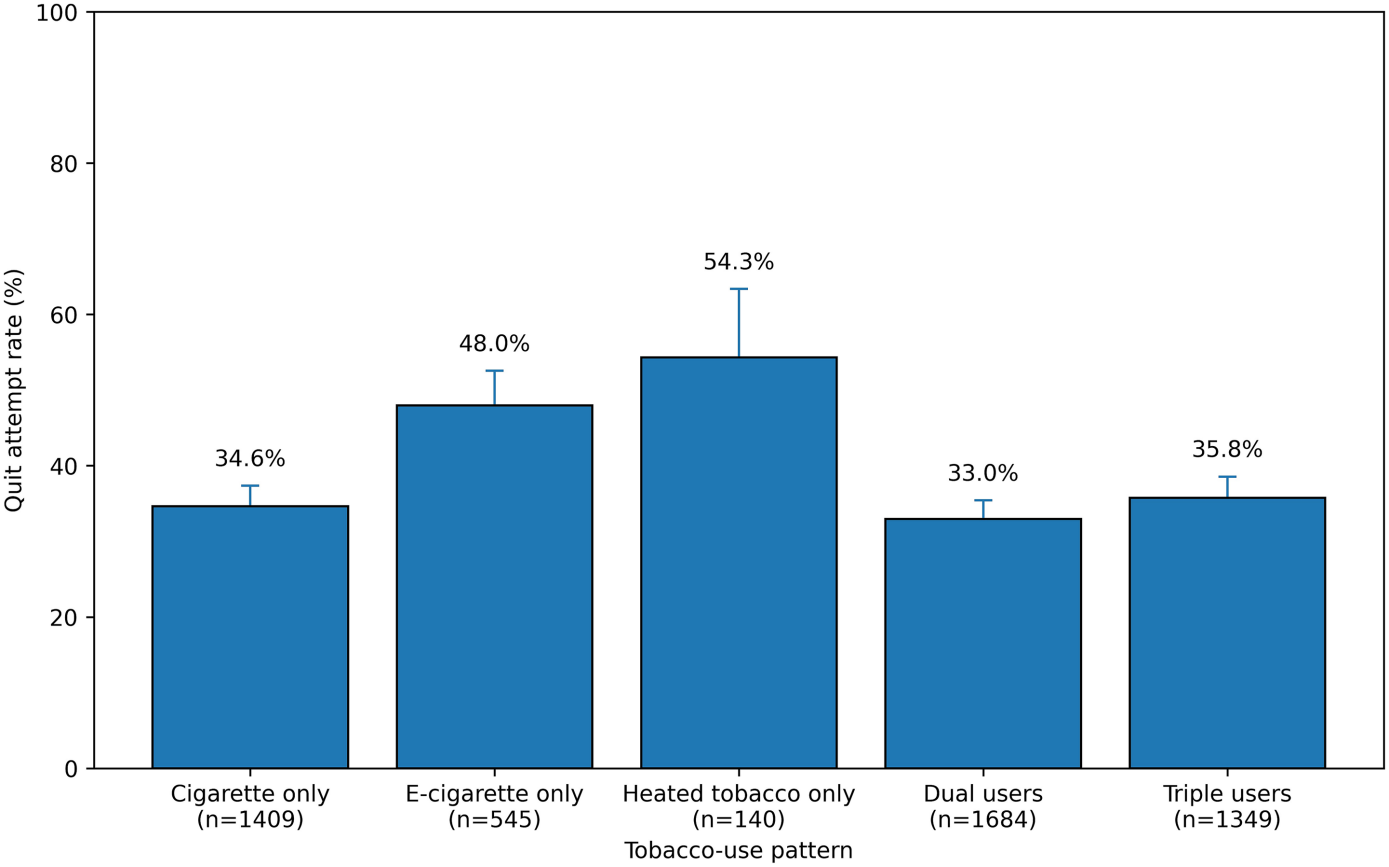
Quit attempt rate by tobacco-use pattern. Weighted quit-attempt rates (past 12 months) are shown across tobacco-use patterns among current adolescent smokers in the pooled 2023–2024 KYRBS sample. Bars represent weighted proportions (%), and error bars indicate 95% confidence intervals. Estimates incorporate sampling weights; when the primary sampling unit (PSU) variable was available, uncertainty was computed using PSU cluster-robust variance from an intercept-only weighted model. When PSU information was unavailable, 95% confidence intervals were approximated using Kish’s effective sample size. Unweighted sample sizes (*n*) are displayed beneath each category label. Quit attempt was defined as a self-reported attempt to quit smoking within the past 12 months.

Among poly-tobacco users, the concurrent use of all three product types (CCs, ECs, and HTPs) was the most prevalent combination, accounting for 44.2% of this group. The dual use of CCs and ECs was the second most common pattern at 35.4% (Supplementary Figure 1). Rates of quit attempts varied markedly by the specific type of tobacco product used. Poly-tobacco users (dual: 33.0%; triple: 35.8%) and single CC users (34.6%) reported the lowest rates of quit attempts. In contrast, adolescents who exclusively used HTPs reported the highest rate of quit attempts at 54.3%, a finding that suggests motivations and barriers for quitting may differ substantially across product-specific user groups (Figure 1).

### Multi-level predictors of quit attempts in the full sample

The hierarchical logistic regression analysis, which included the entire sample of smokers, identified significant predictors of quit attempts across multiple socio-ecological levels (Table 2). The final model (Model 3), which incorporated variables from all levels, demonstrated the best fit and explanatory power. At the individual level, students in their final year of high school and female students were significantly less likely to have made a quit attempt. In contrast, at the interpersonal and organizational levels, having friends who smoke and receiving school-based smoking prevention education were identified as significant facilitators of quit attempts (Table 2). Supplementary Figure 2 conceptually summarizes this multi-level influence.

**Table 2:** Hierarchical logistic regression of multi-level predictors of quit attempts.

Predictor	Model 1		Model 2		Model 3		Model 4	
	aOR (95% CI)	*p*-value	aOR (95% CI)	*p*-value	aOR (95% CI)	*p*-value	aOR (95% CI)	*p*-value
Model 1: individual factors								
6th Grade (vs. 1st)	0.65 (0.46–0.93)	0.018	0.62 (0.43–0.88)	0.007	0.60 (0.42–0.85)	0.004	0.60 (0.42–0.85)	0.005
Sex (Female vs. Male)	0.83 (0.73–0.96)	0.011	0.84 (0.73–0.96)	0.013	0.84 (0.73–0.97)	0.019	0.85 (0.73–0.97)	0.021
Low academic performance	1.14 (1.01–1.30)	0.033	1.14 (1.00–1.29)	0.042	1.13 (0.99–1.28)	0.063	1.13 (0.99–1.28)	0.066
Depressive feelings	1.02 (0.89–1.16)	0.829	1.02 (0.89–1.16)	0.822	1.00 (0.87–1.15)	0.979	0.94 (0.77–1.16)	0.576
High perceived stress	1.00 (0.87–1.14)	0.956	0.98 (0.86–1.13)	0.804	1.00 (0.87–1.15)	0.980	1.03 (0.82–1.28)	0.813
Poly-tobacco user	1.24 (1.10–1.41)	<0.001	1.24 (1.09–1.40)	0.001	1.19 (1.05–1.36)	0.008	1.44 (1.10–1.89)	0.008
Model 2: interpersonal factors								
Family smoker			1.13 (0.94–1.36)	0.197	1.20 (0.99–1.46)	0.059	1.22 (1.01–1.48)	0.042
Friend smoker			1.23 (1.03–1.48)	0.025	1.95 (1.42–2.67)	<0.001	2.21 (1.44–3.39)	<0.001
Home secondhand smoke			0.87 (0.76–0.99)	0.038	0.87 (0.76–0.99)	0.038	1.02 (0.82–1.26)	0.873
Model 3: organizational/policy								
Easy access to tobacco					1.08 (0.94–1.24)	0.265	1.53 (1.23–1.90)	<0.001
School-based education					1.60 (1.30–1.96)	<0.001	1.53 (1.14–2.04)	0.004
Anti-tobacco media					0.42 (0.30–0.58)	<0.001	0.36 (0.23–0.55)	<0.001
Model 4: interaction terms								
Poly-user × Easy access							0.58 (0.45–0.77)	<0.001
Poly-user × Education							1.09 (0.73–1.61)	0.677
Poly-user × Friend smoker							0.68 (0.34–1.37)	0.283
Poly-user × Home SHS							0.77 (0.58–1.02)	0.072
Poly-user × Media							1.53 (0.78–2.99)	0.217
Poly-user × Depressive							1.12 (0.85–1.46)	0.422
Poly-user × High stress							0.94 (0.72–1.25)	0.690

aOR = adjusted odds ratio; CI = confidence interval. Model 1: individual-level; Model 2: +interpersonal; Model 3: +organizational/policy; Model 4: +interaction terms.

### Stratified analyses: common and divergent predictors by user type

**Figure 2:**
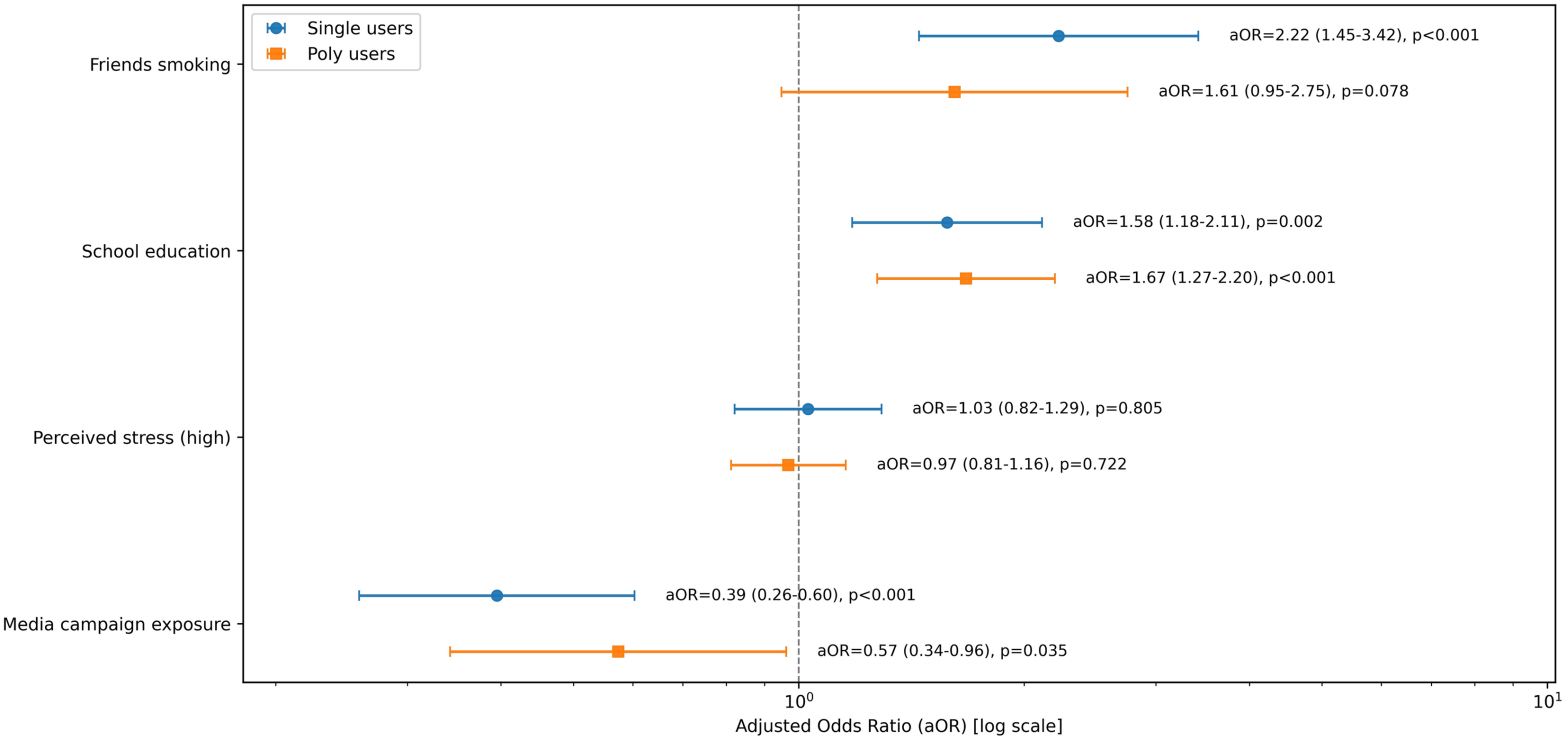
Predictors of quit attempts: single vs poly-product users. Forest plot of adjusted odds ratios (aORs) and 95% confidence intervals (CIs) from weighted logistic regression models stratified by user type (single-product vs. poly-tobacco users). The outcome was quit attempt within the past 12 months (yes/no). Models included the listed key predictors simultaneously and adjusted for available covariates (e.g., sex, grade, and other socio-ecological factors as specified in the Methods). Sampling weights were applied when available; standard errors were estimated using cluster-robust variance at the primary sampling unit (PSU/cluster) level when such variables were available, otherwise using robust (HC1) variance. Points indicate aORs and horizontal lines indicate 95% CIs; the vertical dashed line denotes aOR = 1.0. Exact aOR (95% CI) and *p*-values are annotated to the right of each estimate.

To investigate whether the effects of these predictors were consistent across user types, stratified logistic regression analyses were conducted (Figure 2). Several factors emerged as common predictors for both groups. Exposure to school-based smoking prevention education was positively associated with quit attempts for both single-product users (aOR = 1.35, 95% CI [1.09–1.67]) and poly-tobacco users (aOR = 1.56, 95% CI [1.30–1.88]). Similarly, having friends who smoke was, perhaps counterintuitively, associated with increased odds of attempting to quit in both groups (single users: aOR = 1.28, 95% CI [1.04–1.58]; poly-users: aOR = 1.35, 95% CI [1.14–1.61]), suggesting that peer influence may function in complex ways, potentially increasing exposure to cessation-related conversations or social pressures.

Despite these commonalities, the stratified analyses also highlighted critical differences in predictors between the two groups. Exposure to anti-smoking media campaigns was a significant facilitator of quit attempts only among poly-tobacco users (aOR = 1.29, 95% CI [1.09–1.52]). For single-product users, this factor showed no statistically significant effect (aOR = 1.07, 95% CI [0.87–1.31]). This finding suggests that public health messaging may impact adolescents with varied tobacco use patterns differently. Null findings are important; individual-level psychological factors such as high perceived stress were not significantly associated with quit attempts in either group after controlling for other variables.

### Moderating effect of user type on school-based education

**Figure 3:**
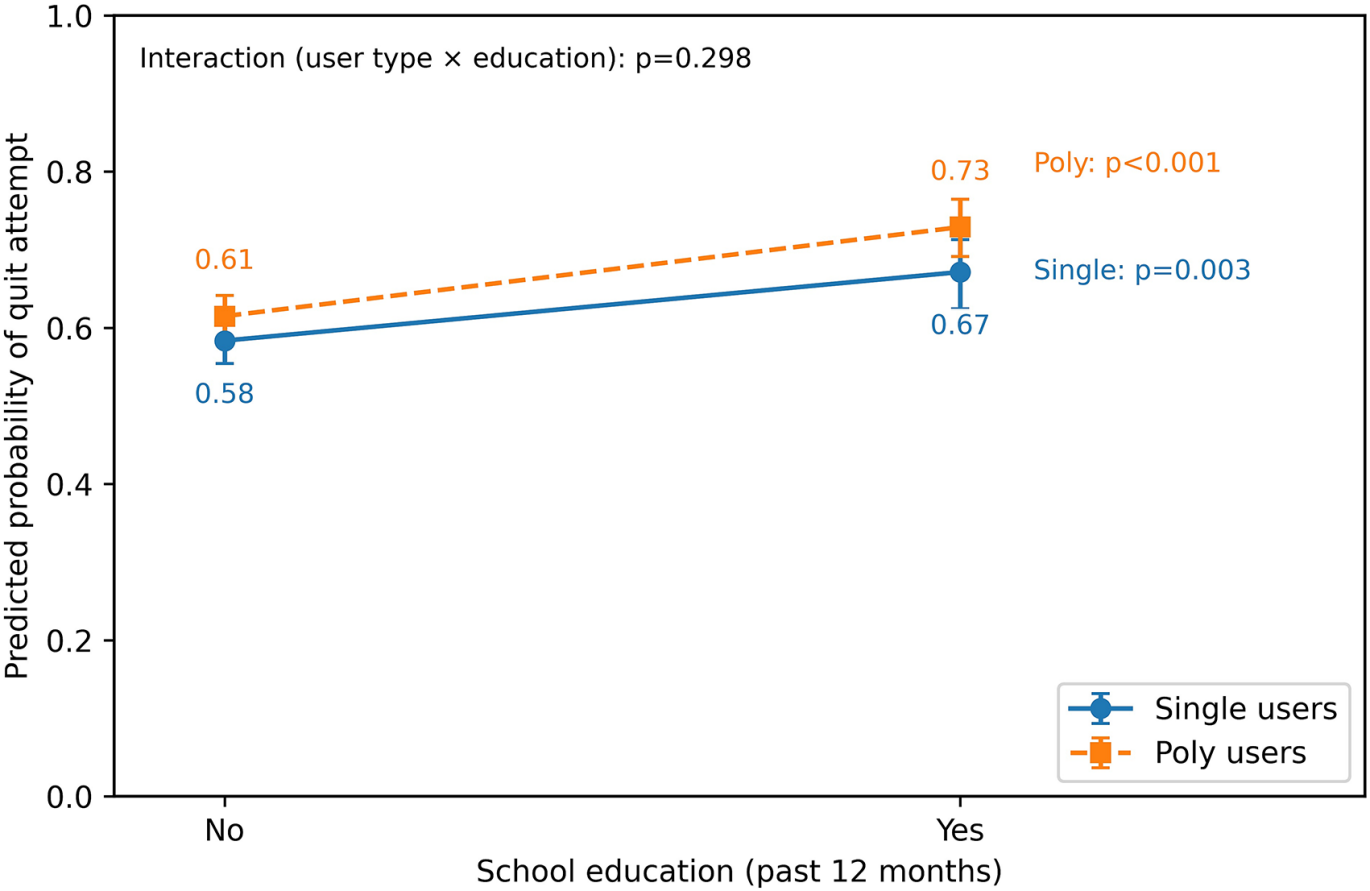
Moderation of school education on quit attempts. Model-based predicted probabilities of a quit attempt (past 12 months) are shown by school-based smoking prevention education (no vs. yes) and tobacco user type (single-product vs. poly-tobacco). Probabilities were derived from a weighted logistic regression model including the interaction term (user type × education) and adjusting for available covariates specified in the Methods. Sampling weights were applied when available; standard errors were estimated using cluster-robust variance at the PSU/cluster level when available, otherwise robust (HC1) variance. Error bars indicate 95% confidence intervals obtained via parametric simulation from the estimated coefficient covariance matrix. *p*-values denote the simple effect of education within each user type (Wald tests), and the interaction *p*-value is reported on the figure.

The interaction analysis formally confirmed that tobacco user type significantly moderated the effect of school-based smoking prevention education on quit attempts (Table 2, Model 4). Figure 3 presents this interaction. While school education was associated with a higher predicted probability of a quit attempt for both groups, the magnitude of this effect was substantially greater for poly-tobacco users. Among students who received no school education, the predicted probabilities of a quit attempt were similar between single- (0.58) and poly-tobacco users (0.61). However, among those who did receive education, the probability increased to 0.67 for single users (a 9-percentage point increase, *p* = 0.003), but rose more sharply to 0.73 for poly-tobacco users (a 12-percentage point increase, *p* < 0.001). This demonstrates a stronger positive impact of educational interventions on the higher-risk group of poly-tobacco users.

## Discussion

This study, utilizing large-scale, nationally representative data from Korean adolescents, provides a nuanced understanding of the factors associated with smoking quit attempts in the contemporary era of poly-tobacco use. Our findings confirm that the adolescent smoking population is not monolithic; single-product and poly-tobacco users, who constitute the majority of current smokers, exhibit distinct characteristics and respond differently to socio-ecological influences. Interpersonal and organizational factors, including peer smoking and school-based education, were linked to quit attempts among all smokers, while community-level factors like media campaigns specifically affected poly-tobacco users. Most notably, this study reveals that school-based education strongly correlates with quit attempts among poly-tobacco users. These findings underscore the critical importance of moving beyond a one-size-fits-all approach and developing tailored tobacco control strategies for different adolescent user groups.

Our findings demonstrate that factors across multiple levels of the socio-ecological model independently and interactively influence quit attempts among adolescent smokers. At the individual level, although poly-tobacco users exhibited a more vulnerable profile, psychological factors such as perceived stress and academic performance did not emerge as significant predictors in the fully adjusted model. This suggests that interpersonal and organizational influences may exert a more dominant role than internal states in shaping quit attempt behaviors among Korean youth.

This study’s results both align with and extend the existing literature on adolescent smoking cessation. The finding that school-based smoking prevention education is positively associated with quit attempts is consistent with previous studies demonstrating the general effectiveness of such programs in improving knowledge, attitudes, and sometimes behavior (29, 30). While some reviews have noted inconsistent results and challenges in achieving long-term effects, our study contributes to the evidence base supporting schools as a vital setting for tobacco control interventions (30, 31). The counterintuitive finding that having friends who smoke was associated with a higher likelihood of a quit attempt contrasts with the bulk of literature, which identifies peer smoking as a primary risk factor for initiation and a barrier to successful cessation (32, 33).

However, this study highlights the distinction between quit attempts and quit success. It is plausible that adolescents within smoking peer groups are more frequently exposed to cessation-related cues, such as friends attempting to quit, discussions about the negative effects of smoking, or shared desires to stop, which may trigger more frequent, albeit potentially less successful, quit attempts. This aligns with social network theories suggesting that health behaviors, including cessation, can spread through peer connections, making the social environment a complex nexus of both risk and opportunity (34, 35). Notably, the paradoxical finding that poly-tobacco users report more quit attempts may be linked to their motivations for initiating novel products. In South Korea, many adolescents perceive ECs and HTPs as “harm-reduction” tools or bridge products to quit CCs, influenced by aggressive marketing that emphasizes reduced odor and perceived safety. Consequently, for many Korean youth, poly-tobacco use may not represent an escalation of nicotine intake alone, but rather a misinformed attempt to transition away from conventional smoking. This interpretation suggests that their higher rate of quit attempts reflects a high level of cessation interest that is currently being channeled into ineffective multiple product use rather than evidence-based cessation methods.

The differential impact of predictors across user types is a main finding of this study. The finding that only poly-tobacco users significantly associated anti-smoking media campaigns with quit attempts is particularly salient. This suggests that adolescents engaged in more complex, high-risk tobacco use may be more receptive to broad, population-level public health messaging. Previous research has shown that mass media campaigns, particularly those with high intensity and duration, can effectively reduce smoking behaviors among youth (36, 37). Poly-tobacco users, who are often more deeply embedded in tobacco product culture and exposed to diverse marketing, may be more attuned to counter-marketing messages that challenge industry tactics or highlight severe health consequences (38–40). This receptivity among high-risk youth is supported by recent evidence (41), who demonstrated that the effectiveness of anti-vaping health communication campaigns varies significantly across different segments of the student population, with specific messaging being more effective for those already engaged in diverse tobacco use. However, given the cross-sectional nature of our study, these findings should be interpreted with caution. The observed associations suggest potential receptivity rather than a direct causal response to the campaigns themselves. In contrast, our null finding regarding the association between perceived stress and quit attempts in the fully adjusted model differs from studies that identify stress as a significant barrier to cessation (42). This discrepancy suggests that in a comprehensive socio-ecological model, the influence of individual psychological states such as stress is mediated or overshadowed by closer social and environmental factors.

When comparing these patterns to global trends, the Korean context displays unique characteristics. While the United States and many European countries have seen an “e-cigarette epidemic” among youth, Korea has experienced a rapid simultaneous uptake of HTPs alongside ECs. This study finds that exclusive HTP users had the highest quit attempt rate (54.3%) mirrors some global observations where HTPs are marketed as high-tech, cleaner alternatives, yet the prevalence of triple-product use remains higher in Korea than in many Western counterparts. This suggests that while the motivation to quit via novel products is a global phenomenon, the specific trajectory of poly-tobacco use in Korea is shaped by a domestic market that rapidly adopts diverse technologies. Our findings underscore that adolescent poly-tobacco use is a cross-cultural challenge, but one that requires regionally tailored messaging to debunk the “healthier alternative” myth prevalent in the Korean market.

This study makes several contributions to the field of adolescent tobacco control. Its primary innovation is the direct, stratified comparison of cessation predictors between single- and poly-tobacco users within a large, nationally representative sample, addressing a critical gap in literature that has historically treated adolescent smokers as a homogenous group. The most significant finding is that school-based education has a stronger positive association with quit attempts among poly-tobacco users. It challenges the assumption that higher-risk users are inherently more resistant to intervention; instead, it suggests they may be a particularly receptive audience for school-based programs, perhaps because their more complex use patterns make the health risks and consequences more salient. This finding provides strong evidence-based rationale for intensifying, rather than abandoning, school-based efforts in the face of the poly-tobacco epidemic. The differential effect of media campaigns further refines this understanding, indicating that a dual strategy—leveraging schools for universal reach and targeted impact and mass media for influencing high-risk users—is likely to be most effective.

This study has several limitations. First, the cross-sectional design of the KYRBS precludes the establishment of causality. Therefore, the identified predictors should be strictly interpreted as statistical correlations rather than causal factors; the observed associations do not prove that educational programs or media campaigns directly cause quit attempts. Longitudinal studies are needed to confirm the temporal sequence of these relationships. Second, all data were based on self-report, which is subject to recall and social desirability biases, though the anonymous nature of the online survey likely mitigates the latter. Third, key variables, such as quit attempts and user type, were based on single items and past 30-day use, which may not fully capture the complexity of cessation journeys or long-term usage patterns. Nicotine dependence, a crucial factor in cessation, was not directly measured. The study focused on Korean adolescents, so the findings may not apply to youth in other countries with different cultures and tobacco control measures.

In conclusion, adolescent quit attempts are not uniform but vary significantly by tobacco use complexity. Our findings offer critical policy implications, suggesting that public health strategies should shift from generic messaging to tailored interventions that address the specific needs of poly-tobacco users. Specifically, enhancing the quality of school-based education and optimizing anti-smoking media campaigns with product-specific messaging could significantly increase quit attempt rates in this vulnerable group. However, the cross-sectional nature of this data limits our ability to infer causal relationships. Future longitudinal research is necessary to investigate the underlying mechanisms of why poly-tobacco users show higher receptivity to institutional support and to determine the long-term success of these quit attempts.

## Data Availability

The data analyzed in this study is subject to the following licenses/restrictions: the data that support the findings of this study are available in Korea Youth Risk Behavior Survey at https://www.kdca.go.kr/yhs/. Requests to access these datasets should be directed to https://www.kdca.go.kr/yhs/.

## Supplementary material

SUPPLEMENTARY FIGURE 1 Distribution of product combinations among poly-tobacco users.

SUPPLEMENTARY FIGURE 2 Conceptual model of the final socio-ecological analysis.
