# Supplementary figures and images for: Differential predictors of smoking quit attempts between single- and poly-tobacco-using adolescents: a Korean national survey analysis
Source: Front Public Health. 2026 Apr 7;14:1727685. doi: 10.3389/fpubh.2026.1727685 (PMC13108398; doi:10.3389/fpubh.2026.1727685)

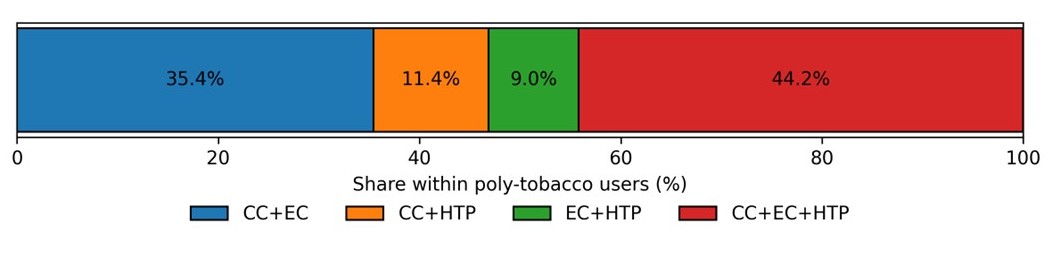

Supplement: SUPPLEMENTARY FIGURE 1 — Distribution of product combinations among poly-tobacco users. [file Image_1.JPEG]

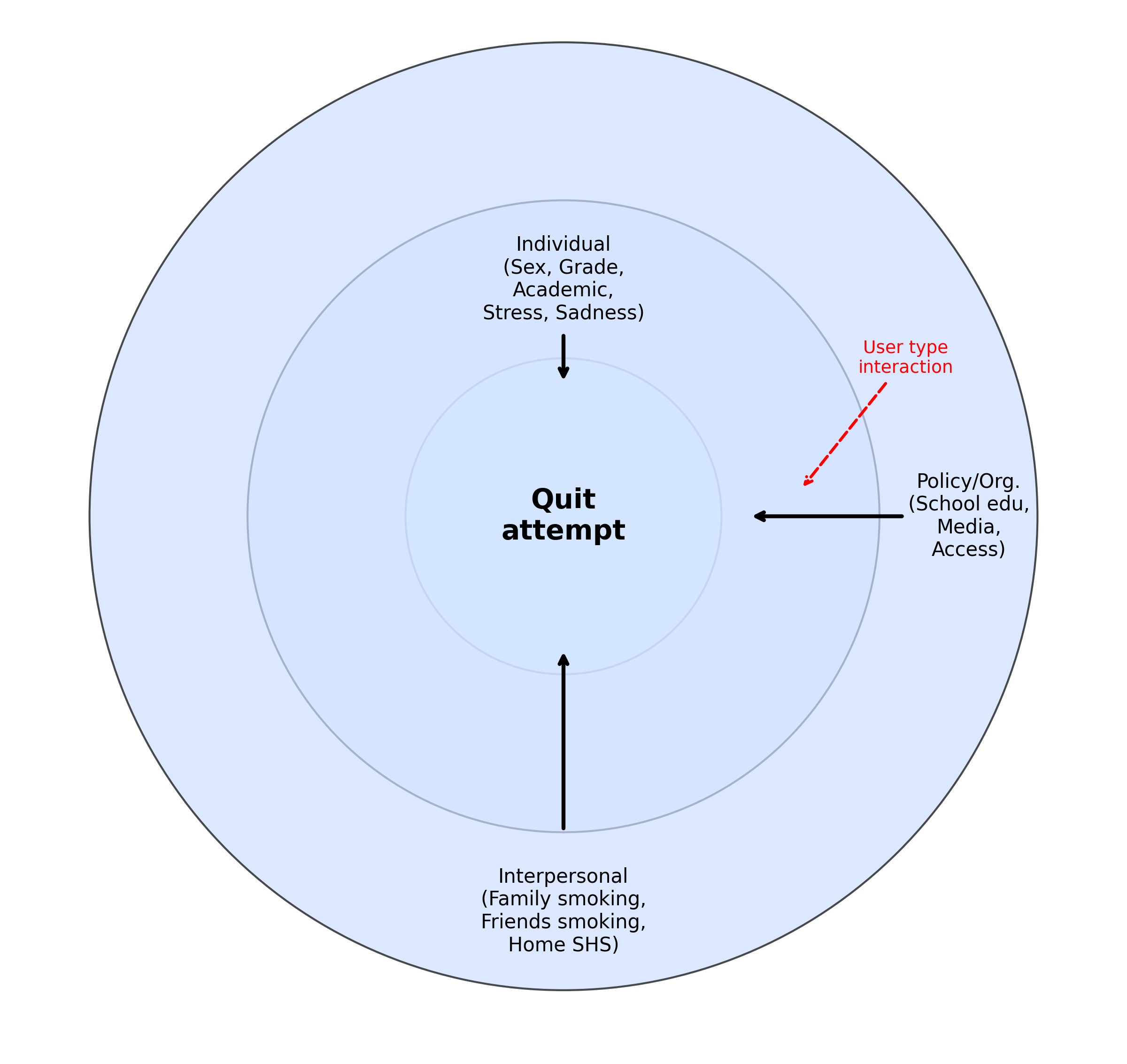

Supplement: SUPPLEMENTARY FIGURE 2 — Conceptual model of the final socio-ecological analysis. [file Image_2.JPEG]
